# New Avian Hepadnavirus in Palaeognathous Bird, Germany

**DOI:** 10.3201/eid2312.161634

**Published:** 2017-12

**Authors:** Wendy K. Jo, Vanessa M. Pfankuche, Henning Petersen, Samuel Frei, Maya Kummrow, Stephan Lorenzen, Martin Ludlow, Julia Metzger, Wolfgang Baumgärtner, Albert Osterhaus, Erhard van der Vries

**Affiliations:** University of Veterinary Medicine Hannover, Foundation, Hannover, Germany (W.K. Jo, V.M. Pfankuche, H. Petersen, M. Ludlow, J. Metzger, W. Baumgärtner, A. Osterhaus, E. van der Vries);; Center for Systems Neuroscience, Hannover (W.K. Jo, V.M. Pfankuche, W. Baumgärtner, A. Osterhaus);; Wuppertal Zoo, Wuppertal, Germany (S. Frei, M. Kummrow);; Bernhard Nocht Institute for Tropical Medicine, Hamburg (S. Lorenzen);; Artemis One Health, Utrecht, the Netherlands (A. Osterhaus)

**Keywords:** HBV, hepatitis B virus, avian hepadnavirus, elegant-crested tinamou, palaeognathae, viruses, Germany, the Netherlands

## Abstract

In 2015, we identified an avian hepatitis B virus associated with hepatitis in a group of captive elegant-crested tinamous (*Eudromia elegans*) in Germany. The full-length genome of this virus shares <76% sequence identity with other avihepadnaviruses. The virus may therefore be considered a new extant avian hepadnavirus.

Hepatitis B virus (HBV) belongs to the family *Hepadnaviridae*, members of which constitute 2 major extant genera: *Orthohepadnavirus*, which infect mammals, and *Avihepadnavirus*, which infect birds (*1*). Recently, evidence of a likely third genus was found with the discovery of a new fish hepadnavirus (*2*). In addition, HBV-derived endogenous viral elements have been reported in several neoavian birds (e.g., budgerigars and several finches) (*3**,**4*) and reptiles (e.g., turtles and crocodiles) (*5*). 

Hepadnaviruses generally are characterized by their narrow host range and strong hepatotropism (*1*). They are enveloped, partially double-stranded DNA viruses with a small circular genome (≈3 kb) and at least 3 open reading frames (ORFs) (*1*). In orthohepadnaviruses, a fourth ORF encodes the X protein, which is associated with hepatocellular carcinoma in their respective host species. Avihepadnaviruses appear to have an X-like protein region; however, either a premature stop codon is present or no ORF is found in most cases (*6*). We describe a new avian HBV causing severe hepatitis in the elegant-crested tinamou (*Eudromia elegans*), a member of the ancient group of birds the Palaeognathae, which includes emus (*Dromaius novaehollandiae*) and ostriches (*Struthio* spp.).

In 2015, a deceased adult elegant-crested tinamou kept at Wuppertal Zoo (Wuppertal, Germany) underwent necropsy at the University of Veterinary Medicine Hannover, Foundation (Hannover, Germany). Initial histologic examination revealed moderate, necrotizing hepatitis and inclusion body–like structures within the hepatocytes. To identify a putative causative agent, we isolated nucleic acids from the liver and prepared them for sequencing on an Illumina MiSeq system (Illumina, San Diego, CA, USA) (Technical Appendix). We compared obtained reads with sequences in GenBank using an in-house metagenomics pipeline. Approximately 78% of the reads aligned to existing avihepadnavirus sequences. A full genome (3,024 bp) of the putative elegant-crested tinamou HBV (ETHBV) was subsequently constructed by de novo assembly mapping >2 million reads (88.6%) to the virus genome (GenBank accession no. KY977506).

The newly identified ETHBV shared <76% nt sequence identity with other avian HBVs (Technical Appendix Table 1). Phylogenetic analysis showed that ETHBV clustered within the genus *Avihepadnavirus*, forming a new clade (Figure, panel A). The organization of the ETHBV genome was similar to other avian HBVs because all 3 overlapping ORFs (polymerase, nucleocapsid [preC/C] and presurface [preS/S] antigen) and several essential sequence motifs (e.g., the epsilon element, TATA boxes, and direct repeat sites DR1 and DR2) were identified (Technical Appendix Figure 1). We also found an X-like sequence. However, similar to duck HBV, ETHBV lacks a putative translation start site. It has been suggested that the X protein evolved later in mammalian hosts (*5*), which explains the absence of X-like ORF in the ETHBV genome. Comparison of pairwise amino acid identities between ETHBV and other avihepadnaviruses showed low homologies between their functional proteins (64%–69% similarity to the polymerase, 75%–80% to the preC/C, 52%–62% to the preS/S [Technical Appendix Table 2]).

**Figure:**
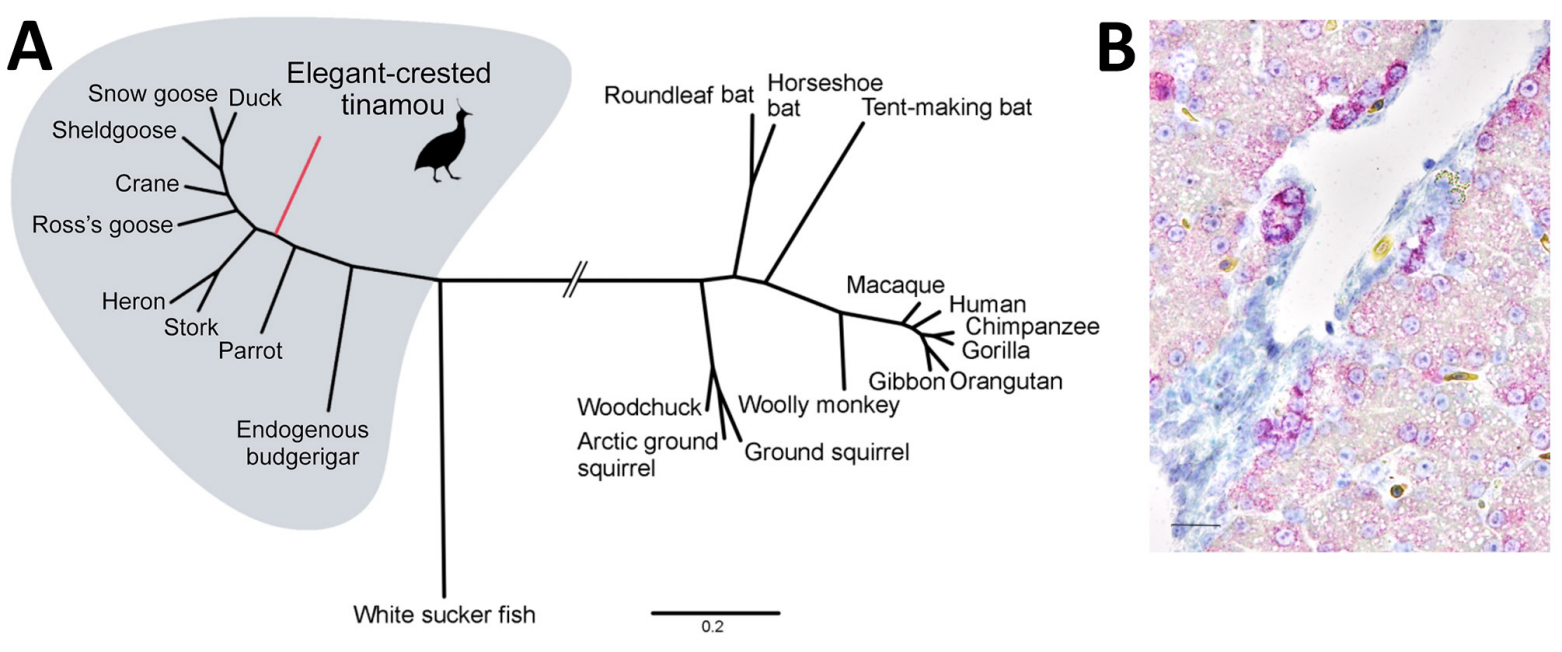
Phylogenetic and histopathologic analysis of probable new avian hepadnavirus, elegant-crested tinamou hepatitis B virus (ETHBV), Germany. A) Bayesian phylogeny of virus isolated from an elegant-crested tinamou (*Eudromia elegans*) compared with reference viruses. Tree was created on the basis of full-genome sequences from the family *Hepadnaviridae*. The analysis was run for 4 million generations and sampled every 100 steps, and the first 25% of samples were discarded as burn-in in MrBayes (*7*). Hasegawa-Kishino-Yano nucleotide substitution model was selected as best-fit model according to Bayesian information criteria. Posterior probabilities are shown in Technical Appendix Figure 3. Branches were truncated for graphical reasons (interrupted lines). Scale bar indicates nucleotide substitutions per site. . GenBank accession numbers are provided online (https://wwwnc.cdc.gov/EID/article/23/12/16-1634-F1.htm). B) ETHBV-specific RNA (in red; Fast Red) localized within hepatocytes of the liver tissue of an elegant-crested tinamou embryo by in situ hybridization (Technical Appendix). Positive signal is enhanced in hepatocytes localized close to the vessels and negative in endothelial cells. Nonprobe incubation of the tinamou and liver tissue from a pheasant were used as negative controls. Scale bar indicates 40 μm.

The identification of ETHBV prompted us to retrospectively screen the flock of 7 elegant-crested tinamous at Wuppertal Zoo and the 6 that had died within the past 4 years and had undergone necropsy at the University of Veterinary Medicine Hannover, Foundation (Technical Appendix Table 4). For that purpose, we designed a set of degenerated primers targeting a short region of the polymerase–preC/C genome in all avihepadnaviruses (Technical Appendix). All birds were found positive by PCR (Technical Appendix Table 4), including liver tissue from embryonated eggs, implying that ETHBV is vertically transmitted (Figure, panel B). We then obtained a second ETHBV genome (GenBank accession no. KY977507) from another tinamou from the same flock by deep sequencing; this genome showed 99.8% nt sequence identity with the initial ETHBV genome. Tinamou serum samples from another zoo were also screened but tested negative by PCR (Technical Appendix Table 4).

To further characterize ETHBV, we confirmed infection in the liver using an in situ hybridization protocol (*8*) in an adult and embryo tinamou (Figure, panel B). In addition to ETHBV infection in the liver, we found some positive cells in kidney and testis tissue. Although hepadnaviruses generally are host restricted, exceptions have been reported (e.g., crane HBV) (*9*). We attempted to infect Pekin duck embryos through the allantoic cavity, as well as by intravenous infection routes, and were not able to demonstrate replication (data not shown).

ETHBV can be considered a new extant hepadnavirus associated with hepatitis in the elegant-crested tinamou. Whether ETHBV can infect other species within the Palaeognathae or whether it is host restricted within other tinamou species remains to be elucidated. The discovery of ETHBV suggests that other avian species may harbor as-yet undiscovered HBVs. The pathogenesis of avian hepadnavirus infections and the mechanisms of virus transmission in captive tinamou flocks warrant further investigation.

Technical Appendix Additional materials and methods for analysis of probable new avian hepadnavirus in an elegant-crested tinamou, Germany.
