# Supplementary material for: New Avian Hepadnavirus in Palaeognathous Bird, Germany
Source: Emerg Infect Dis. 2017 Dec;23(12):2089–91. doi: 10.3201/eid2312.161634 (PMC5708223; doi:10.3201/eid2312.161634)
Supplement: Technical Appendix — Additional materials and methods for analysis of probable new avian hepadnavirus in an elegant-crested tinamou, Germany. [file 16-1634-Techapp-s1.pdf]

Article DOI: https://doi.org/10.3201/eid2312.161634

New Avian Hepadnavirus in Palaeognathous Bird, Germany

Technical Appendix

Sample Preparation and Sequencing

Virus enrichment procedures were applied to the liver tissue before DNA and RNA extractions. Briefly, after homogenization, 3 cycles of freeze/thaw were applied, followed by host nucleic acids degradation with OmniCleave endonuclease (Epicenter Biotechnologies, Madison, WI, USA). RNA and DNA were extracted following TRIzol and the QIAamp DNA mini Kit (Qiagen, Hilden, Germany) procedures, respectively. Next, nucleic acids were randomly amplified using a modified sequence-independent single-primer amplification protocol (*1*) in which random hexamers were replaced by nonribosomal hexamers (*2*). Library preparation was performed following Nextera XT DNA Sample Preparation Kit protocol (Illumina, San Diego, CA, USA). Samples were then sequenced on an Illumina MiSeq sequencer with the MiSeq Reagent Kit v3 (300 × 2 cycles). De novo assembly was performed using the software CLC Genomics Workbench 8.0.3 (https://www.qiagenbioinformatics.com/products/clc-genomics-workbench/).

Detection of Avihepadnaviruses by Quantitative PCR

Oligonucleotides were designed targeting conserved regions between 78 avian hepatitis B viruses (HBVs) aligned genome sequences, including elegant-crested tinamou hepatitis B virus (ETHBV). The resulting forward primer ATTGAAGCAATCACTmGACCAmTCC and reverse primer CTTwGAACGTCTTCTCCCATAGAC amplify a 137-bp fragment within the polymerase and nucleocapsid regions. Quantitative PCR (qPCR) reactions consisted of 1 μL of DNA template, 1× SYBR green mix (Agilent, Santa Clara, CA, USA), 250 nM of forward and reverse primers, making a total of 20 μL reaction volume. The amplification protocol used was as follows: 95°C for 3 min, 40 cycles of 95°C for 10 s, 50°C for 10 s, and 60°C for 10 s in a

qPCR instrument. A melting curve was generated following 1 cycle of 35°C for 30 s, 60°C for 30 s, and 95°C for 30 s. The qPCR sensitivity was evaluated using 10-fold dilution series of ETHBV DNA samples (Technical Appendix Table 3). Moreover, FFPE-positive liver tissues and a negative control (chicken embryo fibroblast cells) samples were also tested.

Amplification of ETHBV Full Genome

Full genome of ETHBV was obtained by conventional PCR using abutting primers (ETHBV_fw: ATGATGCAGGAATCCTTTATAAGCGAG; ETHBV_rv: AAAGCTTAGTTAAATATTCCCCAACTATCTCC) targeting the polymerase region (Technical Appendix Figure 1). PCR reactions consisted of 1 μL of DNA template, 1× buffer, 200 nM dNTPs, 0.5 μM forward and reverse primers, and 1U of Phusion polymerase (New England Biolabs, Ipswich, MA, USA), making 50 μL of total reaction volume. Amplification protocol used was as follows: 98°C for 30 s, 35 cycles of amplification (98°C for 30 s, 64°C for 1 min, and 72°C for 2 min), and a final extension of 72°C for 10 min. A full genome of 3,024 bp was amplified (Technical Appendix Figure 3). Moreover, positive band was cut and purified followed by a digestion with *Eco*RI.

In situ Hybridization

In situ hybridization (ISH) targeting regions of the polymerase and presurface antigen of ETHBV (GenBank accession no. KY977506) was performed on formalin-fixed, paraffin-embedded liver section, according to the manufacturer’s protocol (ViewRNA ISH Tissue 1-Plex Assay Kit and ViewRNA Chromogenic Signal Amplification Kit, Affymetrix-Panomics, Santa Clara, CA, USA) with minor variations, as formerly described (*3*).

GenBank Accession Numbers in the Figure

Elegant-crested tinamou, KY977506; woodchuck, AY334076; woolly monkey, AF046996; gibbon, U46935; orangutan, EU155826; gorilla, FJ798096; chimpanzee, D00220; human, AB775200; macaque, HE815465; horseshoe bat, KC790377; roundleaf bat, KC790373; tent-making bat, KC790378; Arctic ground squirrel, U29144; ground squirrel, K02715;

endogenous budgerigar, BK008521; duck, X60213; snow goose, AF110996; sheldgoose, AY494853; crane, AJ441111; Ross's goose, M95589; parrot, JN565944; heron, M22056; stork, AJ251935.

References

1. Allander T, Tammi MT, Eriksson M, Bjerkner A, Tiveljung-Lindell A, Andersson B. Cloning of a human parvovirus by molecular screening of respiratory tract samples. Proc Natl Acad Sci U S A. 2005;102:12891–6. PubMed http://dx.doi.org/10.1073/pnas.0504666102

2. Endoh D, Mizutani T, Kirisawa R, Maki Y, Saito H, Kon Y, et al. Species-independent detection of RNA virus by representational difference analysis using non-ribosomal hexanucleotides for reverse transcription. Nucleic Acids Res. 2005;33:e65. PubMed http://dx.doi.org/10.1093/nar/gni064

3. Pfankuche VM, Bodewes R, Hahn K, Puff C, Beineke A, Habierski A, et al. Porcine bocavirus infection associated with encephalomyelitis in a pig, Germany. Emerg Infect Dis. 2016;22:1310–2. PubMed http://dx.doi.org/10.3201/eid2207.152049

Technical Appendix Table 1. Full genome nucleotide sequence identity between prototype avianhepadnaviruses and the new elegant-crested tinamou hepatitis B virus*

Bird	GenBank accession no.	Elegant-crested tinamou	Duck	Snow goose	Sheldgoose	Crane	Ross's goose	Heron	Stork	Parrot
Elegant-crested tinamou	KY977506	100								
Duck	X60213	74.0	100							
Snow goose	AF110996	74.4	89.2	100						
Sheldgoose	AY494853	73.4	85.0	84.7	100					
Crane	AJ441111	75.9	83.3	83.2	83.2	100				
Ross's goose	M95589	75.1	81.3	80.3	80.5	84.1	100			
Heron	M22056	72.1	76.1	76.0	75.4	77.0	76.6	100		
Stork	AJ251935	72.6	76.2	76.6	76.5	78.6	77.1	85.3	100	
Parrot	JN565944	71.1	73.8	74.0	73.9	74.9	74.0	73.3	73.5	100

*Sequence identities presented as percentages.

Technical Appendix Table 2. Homologies between the different proteins of prototype avihepadnaviruses and the elegant-crested tinamou hepatitis B virus*

Bird	GenBank accession no.	Polymerase	PreC/C	PreS/S	X-like
Duck	X60213	68	75	60	57
Snow goose	AF110996	69	76	61	61
Sheldgoose	AY494853	68	76	57	64
Crane	AJ441111	68	76	62	65
Ross's goose	M95589	68	76	60	61
Heron	M22056	64	79	60	56
Stork	AJ251935	65	80	59	59
Parrot	JN565944	65	78	52	59

*Homologies between amino acid sequences are presented in percentages. PreC/C, nucleocapsid antigen; PreS/S, presurface antigen.

Technical Appendix Table 3. ETHBV DNA samples for evaluating quantitative PCR using SYBR green probe*

Sample	Cycle threshold	Melting temperature
ETHBV	11	81.0
ETHBV 1 × 10^{-1}	14	81.0
ETHBV 1 × 10^{-2}	18	81.0
ETHBV 1 × 10^{-3}	23	81.0
ETHBV 1 × 10^{-4}	25	81.0
ETHBV FFPE	18	80.5
CEF	>30	75.5
NTC	>30	75.5

*ETHBV, elegant-crested tinamou; FFPE, formalin-fixed paraffin-embedded; CEF, chicken embryo fibroblast cells; NTC, negative template control.

Technical Appendix Table 4. Avihepadnavirus qPCR detection in elegant-crested tinamou samples from Wuppertal Zoo (Germany) and GaiaZOO (Netherlands)*

Identifier	Zoo	Status	Histopathologic diagnosis of liver	Sample	C_t	Tm
101-160014	Wuppertal	Dead (died 05/2015)	Hepatitis, necrotizing, moderate, multifocal with intralesional, intranuclear inclusion body-like structures	Liver	10	81.0
				FFPE-Liver	18	80.5
407-160049	Wuppertal	Dead (died 11/2013)	Hepatitis, lymphohistiocytic, mild, oligofocal	Liver	13	81.0
				Lung	15	81.0
				Kidney	19	81.0
				Brain	20	81.0
407-160050	Wuppertal	Dead (died 06/2015)	Hepatitis, lymphohistiocytic, partially suppurative to pyogranulomatous, moderate, multifocal	Liver	7	81.0
				Lung	17	81.0
				Kidney	12	81.0
				Spleen	16	81.0
				Brain	23	81.0
				Intestines	18	81.0
407-160051	Wuppertal	Dead (died 03/2016)	Hepatocellular necrosis, mild, multifocal; hepatitis, lymphohistiocytic, mild, multifocal; hepatocellular vacuolization, mild, diffuse	Liver	8	81.0
				Lung	13	81.0
				Kidney	14	81.0
				Spleen	16	81.0
407-160052	Wuppertal	Dead (died 04/2016)	Hepatitis, necrotizing, moderate, multifocal with intralesional, intranuclear inclusion body-like structures; hemorrhage, moderate, focal, acute	Liver	7	81.0
				Lung	No C_t	81.0
				Kidney	15	81.0
				Brain	18	81.0
				Muscle	19	81.0
407-160053	Wuppertal	Dead (died 05/2016)	Not done	Egg yolk	16	81.0
				Liver	18	81.0
407-160054	Wuppertal	Dead (died 05/2016)	Not done	Egg yolk	13	81.0
				Liver	9	81.0
407-160056	Wuppertal	Alive	Not applicable	Serum	8	81.0
407-160057	Wuppertal	Alive	Not applicable	Serum	9	81.0
407-160058	Wuppertal	Alive	Not applicable	Serum	10	81.0
407-160059	Wuppertal	Alive	Not applicable	Serum	9	81.0
407-160060	Wuppertal	Alive	Not applicable	Serum	12	81.0
407-160376	Wuppertal	Alive	Not applicable	Serum	9	81.0
407-160377	Wuppertal	Alive	Not applicable	Serum	10	81.0
412-160374	GaiaZOO	Alive	Not applicable	Serum	>30	76.0
412-160375	GaiaZOO	Alive	Not applicable	Serum	>30	76.0
412-160376	GaiaZOO	Alive	Not applicable	Serum	>30	76.0

*qPCR, quantitative PCR; C_t, cycle threshold; Tm, melting temperature; negative: Tm ≠ 81 and C_t >30; positive: Tm = 81 and C_t ≤30.

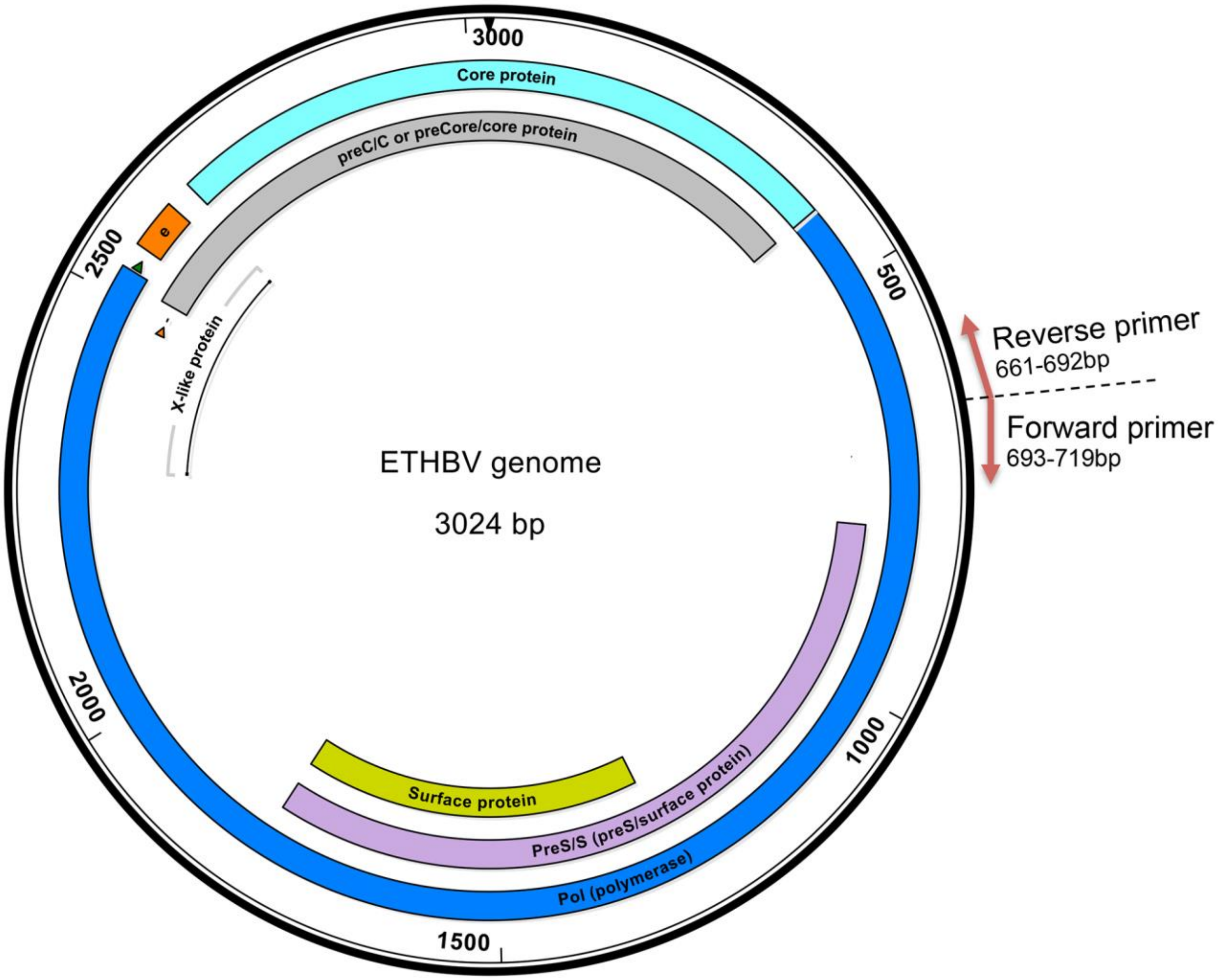

Technical Appendix Figure 1. Abutting primers design for the amplification of ETHBV full-genome representation. The annotated genome contains the coding proteins at the sites 170–2533 bp for the polymerase, 2521–3024, 1–414 bp for the preC/C, 2650–3024, 1–414 bp for the core, 801–1790 bp for the preS/S, and 1290–1790 bp for the surface; an X-like protein within 2292–2636 bp; direct repeat sequences DR1 and DR2 within 2537–2548 bp and 2480–2491 bp, respectively; and the epsilon motive (e) in the region 2563–2619bp. ETHBV, elegant-crested tinamou hepatitis B virus; preC/C, nucleocapsid antigen; preS/S, presurface antigen.

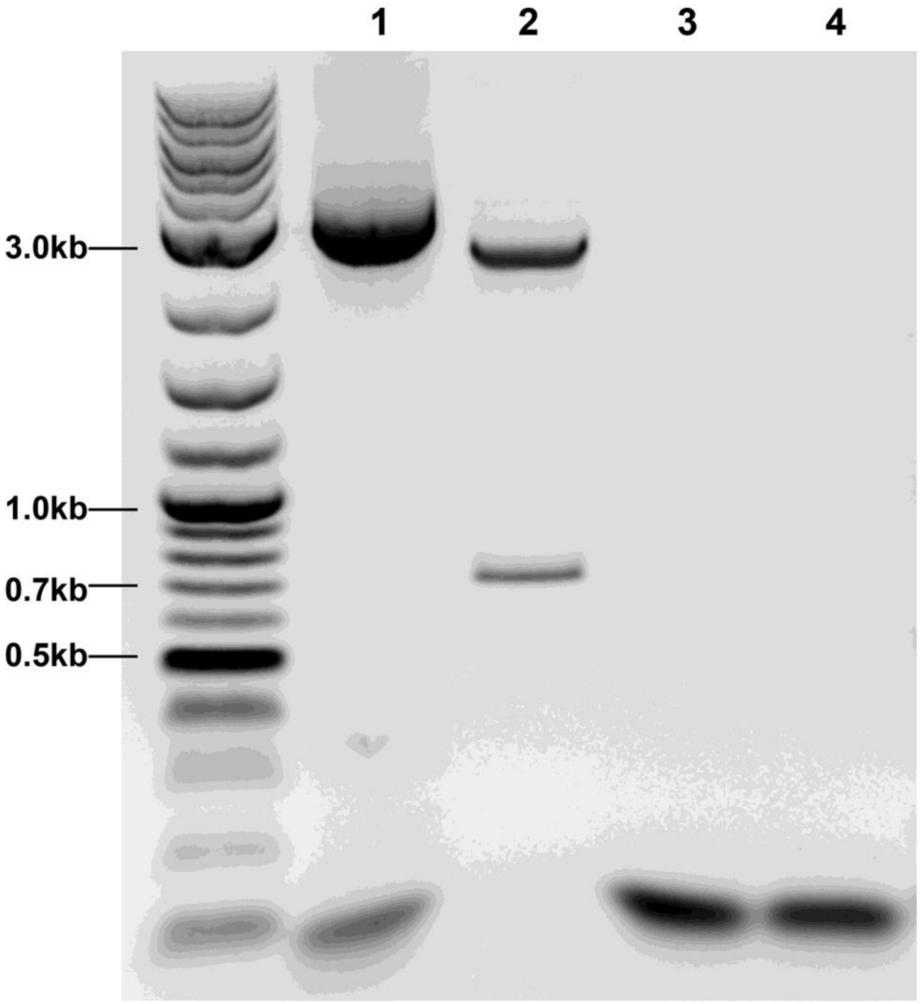

Technical Appendix Figure 2. Elegant-crested tinamou hepatitis B virus (ETHBV) full-genome amplification visualized on a 1% agarose gel. 1) Positive ETHBV sample: expected band at 3024 bp. 2) Digestion of ETHBV with *Eco*RI: expected bands at 696 bp and 2328 bp. 3) Negative control: tinamou serum from GaiaZOO 4) No template control.

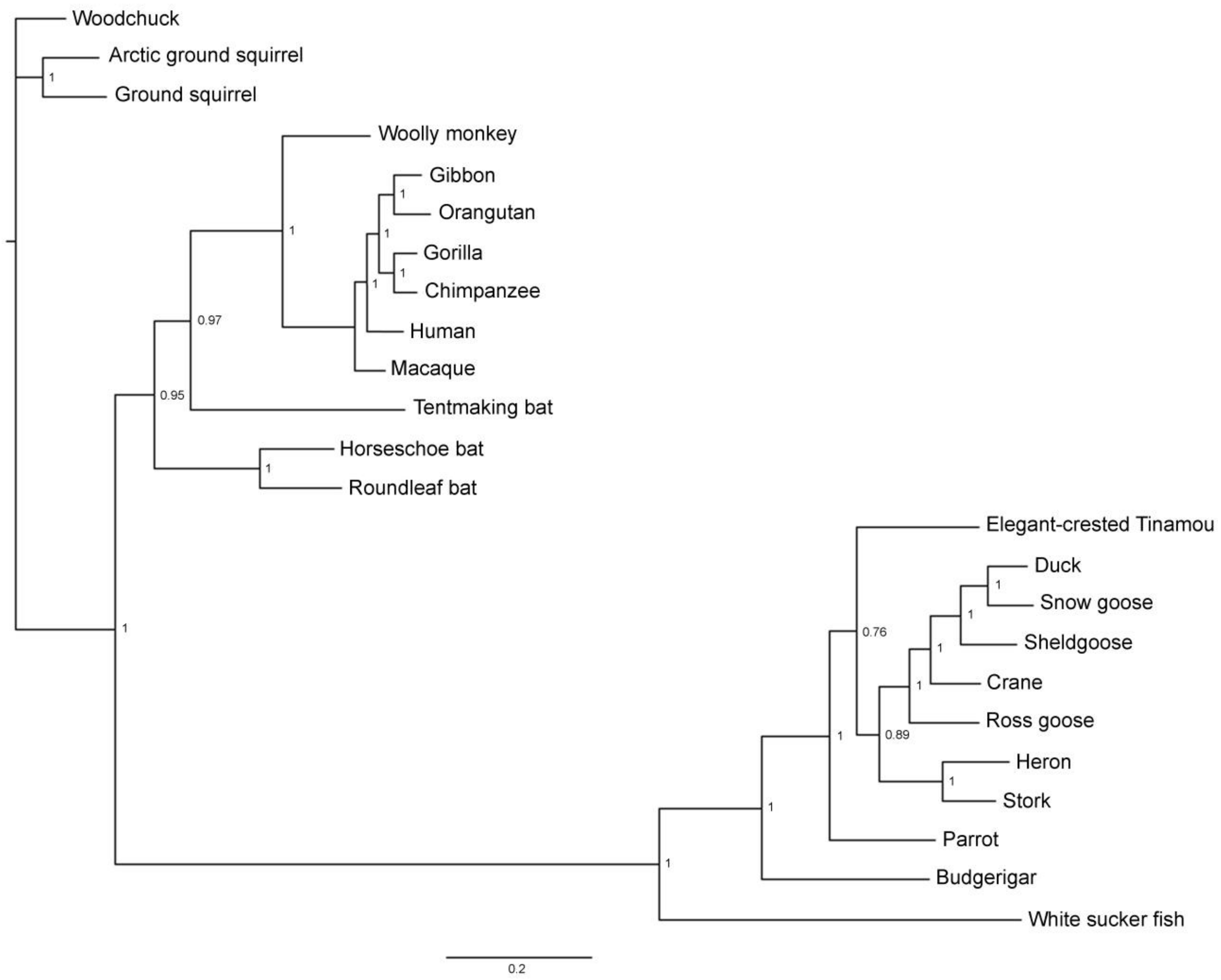

Technical Appendix Figure 3. Bayesian phylogeny with posterior probabilities based on full-genome sequences from the family *Hepadnaviridae*. The analysis was run for 4 million generations and sampled every 100 steps with the first 25% of samples discarded as burn-in in MrBayes. Hasegawa-Kishino-Yano nucleotide substitution model was selected as best-fit model according to Bayesian information criteria. Scale bar indicates nucleotide substitution per site. GenBank accession numbers of hepadnaviruses: woodchuck, AY334076; woolly monkey, AF046996; gibbon, U46935; orangutan, EU155826; gorilla, FJ798096; chimpanzee, D00220; human, AB775200; macaque, HE815465; horseshoe bat, KC790377; roundleaf bat, KC790373; tent-making bat, KC790378; Arctic ground squirrel, U29144; ground squirrel, K02715; endogenous budgerigar, BK008521; duck, X60213; snow goose, AF110996; sheldgoose, AY494853; crane, AJ441111; Ross’s goose, M95589; parrot, JN565944; heron, M22056; stork, AJ251935; elegant-crested tinamou, KY977506.
